# Contemporary analysis of functional immune recovery to opportunistic and vaccine‐preventable infections after allogeneic haemopoietic stem cell transplantation

**DOI:** 10.1002/cti2.1040

**Published:** 2018-10-05

**Authors:** Harini D de Silva, Rosemary A Ffrench, Maya Korem, Eva Orlowski, David J Curtis, Andrew Spencer, Sharon Avery, Sushrut Patil, Catherine Orla Morrissey

**Affiliations:** ^1^ Burnet Institute Life Sciences Discipline Melbourne VIC Australia; ^2^ Department of Infectious Diseases Alfred Health and Monash University Melbourne VIC Australia; ^3^ Department of Immunology Central Clinical School Monash University Melbourne VIC Australia; ^4^ Australian Centre for Blood Diseases Monash University Melbourne VIC Australia; ^5^ Malignant Haematology and Stem Cell Transplantation Service Alfred Health Melbourne VIC Australia; ^6^ Present address: Peter MacCallum Cancer Centre Melbourne VIC Australia; ^7^ Present address: Hadassah University Medical Centre Jerusalem Israel

**Keywords:** allogeneic, *Aspergillus*, T‐cell responses, cytokines, vaccines

## Abstract

**Objectives:**

Infections are a major cause of mortality after allogeneic haemopoietic stem cell transplantation (alloHSCT), and immune recovery is necessary for prevention. Novel transplant procedures have changed the epidemiology of infections but contemporary data on functional immune recovery are limited. In this pilot study, we aimed to measure immune recovery in the current era of alloHSCT.

**Methods:**

Twenty, 13, 11, 9 and 9 alloHSCT recipients had blood collected at baseline (time of conditioning) and 3‐, 6‐, 9‐, and 12‐months post‐alloHSCT, respectively. Clinical data were collected, and immune recovery was measured using immunophenotyping, lymphocyte proliferation, cytokine analysis and antibody isotyping.

**Results:**

Median absolute T‐ and B‐cell counts were below normal from baseline until 9‐ to 12‐months post‐alloHSCT. Median absolute CD4^+^ T‐cell counts recovered at 12‐months post‐alloHSCT. Positive proliferative responses to *Aspergillus*, cytomegalovirus (CMV), Epstein‐Barr virus (EBV), influenza and tetanus antigens were detected from 9 months. IL‐6 was the most abundant cytokine in cell cultures. In cultures stimulated with CMV, EBV, influenza and tetanus peptides, the CD4^+^ T‐cell count correlated with IL‐1β (*P *=* *0.045) and CD8^+^ T‐cell count with IFNγ (*P *=* *0.013) and IL‐1β (*P *=* *0.012). The NK‐cell count correlated with IL‐1β (*P *=* *0.02) and IL‐17a (*P *=* *0.03). Median serum levels of IgG1, IgG2 and IgG3 were normal while IgG4 and IgA were below normal range throughout follow‐up.

**Conclusions:**

This pilot study demonstrates that immune recovery can be measured using CD4^+^ T‐cell counts, *in vitro* antigen stimulation and selected cytokines (IFNγ, IL‐1β, IL‐4, IL‐6, IL‐17, IL‐21, IL‐31) in alloHSCT recipients. While larger studies are required, monitoring immune recovery may have utility in predicting infection risk post‐alloHSCT.

## Introduction

Allogeneic haemopoietic stem cell transplantation (alloHSCT) is routinely used to cure a wide variety of haematological malignancies. The immunosuppression, given during conditioning and for graft‐versus‐host disease (GVHD) prophylaxis, results in delayed immune reconstitution and places alloHSCT recipients at high risk for opportunistic and vaccine‐preventable infections.^1^, ^2^, ^3^, ^4^ Infections are a leading cause of death in patients undergoing alloHSCT.^1^, ^3^ While antimicrobial prophylaxis is effective in reducing morbidity and mortality from infections post‐alloHSCT, it has significant limitations including drug‐related toxicity, emergence of resistance, breakthrough infections and high cost.^5^, ^6^

Currently, risk stratification models are used to guide the timing and duration of antimicrobial prophylaxis.^7^ These are based on clinical factors such as age at time of alloHSCT, underlying disease, prior therapy, conditioning regimen, donor type, degree of matching, stem cell source and presence of GVHD. However, such clinically based models only give a qualitative measure of immune function and consequently lack precision. A strategy which quantifies immune function is urgently needed to more accurately predict risk and guide antimicrobial prophylaxis accordingly. Immune‐based strategies for cytomegalovirus (CMV) have been examined and appear promising for guiding CMV prophylaxis;^8^ however, more broad‐based immune function strategies incorporating other important opportunistic pathogens, such as *Aspergillus* still need to be developed.

The alloHSCT process results in loss of immune memory accumulated from previous vaccinations, and all recipients need to be re‐vaccinated post‐alloHSCT.^9^, ^10^ The new naive T and B cells develop from donor stem cells and require stimulation with vaccine antigens for long‐term protection. For vaccination to be useful post‐alloHSCT, it must occur at a time when the immune system has adequate function to generate a protective response. Therefore, the optimal timing of vaccination becomes a critical balance between obtaining a protective immune response as early as possible to prevent infections and delaying it until functionally effective immune responses can be generated. Current post‐alloHSCT vaccination strategies are based on fixed schedules.^11^ However, it is becoming evident that the timing of vaccination would be more appropriately based on each patient's capacity to respond to vaccine antigens. Evolving data suggest that such immune responses can be measured.^12^, ^13^, ^14^ However, a more detailed analysis is required prior to developing novel vaccine schedules to better guide effective vaccination post‐alloHSCT.

Assays to measure immune function are available including immune cell counts,^15^ subpopulations of natural killer (NK) cells,^16^ composition of memory T‐cell compartments,^17^ cytokine profiling^18^ and cellular proliferation measurement.^19^ Studies performed to examine immune reconstitution post‐alloHSCT are limited by contemporary relevance, number of immune markers and number of pathogens examined, or correlation with clinical outcomes. While many studies have provided evidence for the importance of CD8^+^ T cell‐mediated viral‐specific immune recovery post‐alloHSCT,^20^ the recovery of CD4^+^ T‐cell function is less well understood.

The aim of this study was to perform a contemporary and comprehensive examination of immune reconstitution post‐alloHSCT including CD4^+^ T‐cell function and cytokine profiling.

## Results

### Patient characteristics and clinical outcomes

The baseline characteristics are shown in Table 1. Acute myeloid leukaemia was the most common indication for transplantation (5/20; 25%), and nine patients (45%) received a reduced intensity conditioning (RIC) regimen (Table 1).

**Table 1:** Baseline demographic and clinical characteristics

Characteristics	
Age at transplantation – Median (IQR)	46.5 (35.5–57.0)
Male sex, *n* (%)	13 (65)
Underlying disease, *n* (%)	
Acute myeloid leukaemia	5 (25)
Acute lymphoblastic leukaemia	3 (15)
Chronic myeloid leukaemia	2 (10)
Chronic lymphocytic leukaemia	2 (10)
Myelodysplastic syndrome	1 (5)
Aplastic anaemia	2 (10)
Other^a^	5 (25)
Donor type, *n* (%)	
Sibling	11 (55)
Mismatched related	1 (5)
Matched unrelated	4 (20)
Mismatched unrelated	4 (20)
Conditioning regimen, *n* (%)	
Myeloablative	10 (50)
Reduced intensity	9 (45)
T‐cell depletion	
ATG	8 (40)
Alemtuzumab	4 (20)
Other^b^	1 (5)
Stem cell source, *n* (%)	
Bone marrow	4 (20)
Peripheral blood stem cells	16 (80)
Total body irradiation, *n* (%)	6 (30)
Neutrophil engraftment^c^ – Median (IQR) days	23 (21–27)
CMV status, *n* (%)	
Donor+/Recipient+	7 (35)
Donor−/Recipient+	7 (35)
Donor+/Recipient−	1 (5)
Donor−/Recipient−	5 (25)

CMV, cytomegalovirus; IQR, interquartile range; *n*, number.

a T‐cell lymphoma (1), B‐lymphoblastic leukaemia/lymphoma (1), Follicular non‐Hodgkin lymphoma (2), chronic myelo‐monocytic leukaemia (1).

b Nonmyeloablative.

c Defined as an absolute neutrophil count > 0.5 × 10^9^ L^−1^ sustained over 3 days.

Nineteen patients (95%) had ≥ 1 infection during the 12 months of follow‐up (Table 2). Two (10%) had invasive fungal disease, proven disseminated mucormycosis and probable pulmonary aspergillosis diagnosed at 351 and 21 days, respectively (Table 2). Cytomegalovirus viremia was detected in 7 of the 15 at risk of CMV (donor and/or recipient positive; 47%) at a median of 45 (IQR 19–142) days post‐alloHSCT (Table 2).

**Table 2:** Infectious complications and other clinical outcomes through 1 year of follow‐up

Patient	Age	Sex	Underlying disease	Infections		GVHD		Death	
				Type	Day^a^	Type, grade or extent	Day^a^	Cause	Day^a^
1	64	M	Acute myeloid leukaemia	*Aeromonas sobria* bacteremia	13	Chronic localised	240		
				RSV LRTI	55				
				*Haemophilus influenzae* conjunctivitis	180				
				Disseminated mucormycosis (*Rhizopus microsporus*)	351				
2^b^	55	F	Chronic lymphocytic leukaemia						
3	30	M	T‐cell lymphoma	Norovirus gastroenteritis	5	Acute – Grade III	38		
				Polymicrobial bacteremia	16				
				CMV viremia^c^	34				
				Picornavirus LRTI	271				
4	37	M	Acute lymphoblastic leukaemia	Norovirus gastroenteritis	4			Cardio‐respiratory arrest	25
				*Enterococcus faecium* bacteremia	22				
5	22	F	Aplastic anaemia	CMV viremia^c^	45				
				Influenza B LRTI	98				
6	45	M	B‐lymphoblastic leukaemia/lymphoma	*Clostridium difficile*	4	Acute – Grade II	38	Cardio‐respiratory arrest	54
7	43	F	Acute lymphoblastic leukaemia	CMV viremia^c^	11				
				Hepatitis B^d^	14				
				Polyoma viruria	90				
8	43	M	Chronic myelo‐monocytic leukaemia	*Streptococcus mitis* bacteremia	20				
9	42	F	Acute myeloid leukaemia	*Gemella haemolysans* bacteremia	20	Acute – Grade IV	48	GVHD	97
				CMV disease^c^	66				
				Polymicrobial bacteremia	90				
10	34	M	Aplastic anaemia	Picornavirus URTI	43				
11	62	M	Acute lymphoblastic leukaemia	VRE bacteremia	18	Chronic – Localised	169		
				MSSA bacteremia	21				
				*Ralstonia mannitolilytica* LRTI	27				
				Influenza A LRTI	294				
12	36	F	Acute myeloid leukaemia	Polyoma viruria	44	Acute – Grade II	83	Septicaemia^e^	159
				*Esherichia coli* bacteremia	61				
				CMV viremia^c^	79				
13	21	F	Acute lymphoblastic leukaemia	*Klebsiella pneumoniae* UTI	49				
14	52	M	Myelodysplastic syndrome	MSSA bacteremia	21	Acute – Grade IV	33	Septicaemia^f^	59
				*Pseudomonas aeruginosa* bacteremia	56				
15	63	M	Follicular non‐hodgkin lymphoma	VRE bacteremia	78	Acute – Grade II	64	LRTI	148
				Parainfluenza and *K. pneumoniae* LRTI	110				
16	64	F	Acute myelo‐monocytic leukaemia	CMV disease^c^	142	Acute – Grade III	35	CMV disease	174
17	54	M	Chronic lymphocytic leukaemia	*E. faecium* bacteremia	18	Chronic – Extensive	117		
				Picornavirus URTI	19				
				CMV disease^c^	37				
18	59		Acute myeloid leukaemia	*C. difficile*	11	Acute – Grade I	40		
				HMPV URTI	94				
				Parainfluenza URTI	276				
19	48	M	Follicular non‐hodgkin lymphoma	*E. coli* bacteremia	15			–	–
20	50	M	Acute myeloid leukaemia	Invasive aspergillosis	21			Invasive aspergillosis	26

CMV, cytomegalovirus; GVHD, graft‐versus‐host disease; F, female; HMPV, human meta‐pneumovirus; M, male; LRTI, lower respiratory tract infection; MSSA, methicillin sensitive *Staphylococcus aureus*; RSV, respiratory syncytial virus; URTI, upper respiratory tract infection; VRE, vancomycin resistant enterococci.

a Day of diagnosis after allogeneic haemopoietic stem cell transplantation (HSCT).

b Syngeneic HSCT.

c Blood CMV viral load at first detection: 10 825 IU mL^−1^ (patient 3); 150 IU mL^−1^ (patients 5, 7, 9 and 12); 248 IU mL^−1^ (patient 16); 12 162 IU mL^−1^ (patient 17).

d Hepatitis B viral load = 28 IU mL^−1^; first measurement post‐transplant.

e *Enterococcus faecalis*.

f *Pseudomonas aeruginosa*.

Eight patients (40%) died during the 12 months of follow‐up at a median of 78 (IQR 33–156) days (Table 2) with five of eight deaths infection‐related (62.5%).

### Recovery of immune cell subsets

**Figure 1:**
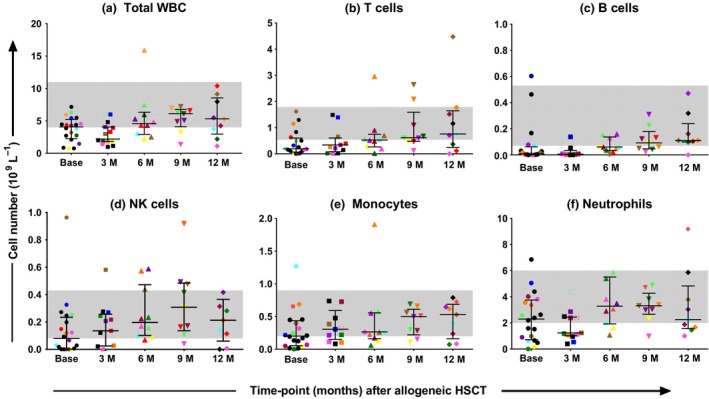
**(a–f)** Recovery of absolute cell numbers of leucocytes (total WBC), T cells, B cells, NK cells, monocytes and neutrophils over 12‐months. HSCT, haemopoietic stem cell transplant; M, month; NK, natural killer; WBC, white blood cell; *N* = 20 at time of conditioning chemotherapy (Base); *N* = 13 at 3 months (3 M); N = 11 at 6 months (6 M) and N = 9 at 9 months (9 M) and 12 months (12 M). The total WBC count was used to calculate absolute numbers for the leucocyte subsets based on flow cytometry immunophenotyping data (percent positive for each subset based on analysis of 100 000 acquired events). The grey band depicts the normal range.^21^ Black horizontal bars represent the median, 25th and 75th percentiles of distribution. Longitudinal samples from each patient are represented by individual colours. Data are representative of one experiment.

Absolute cell numbers for total leucocytes, T, B, NK cells, monocytes and neutrophils are shown in Figure 1a–f. Median absolute T‐ and B‐cells numbers were lowest at baseline (0.19 × 10^9^ L^−1^ and 0.008 × 10^9^ L^−1^, respectively) and increased thereafter, reaching normal range^21^ 9‐ to 12‐months post‐alloHSCT (0.62 × 10^9^ L^−1^ and 0.09 × 10^9^ L^−1^, respectively; Figure 1b, c).

The recovery of percent fractions of each cell subset was also analysed and T‐ and B‐cell percent fractions were seen to recover earlier than absolute cell numbers. The median percent T‐cell values recovered to normal levels 3‐ to 6‐months post‐alloHSCT while median percent B‐cell levels recovered at 6‐ to 9‐months post‐alloHSCT (Supplementary figure 1).

Median absolute NK cells and monocytes were at the lower limit of normal at baseline (0.08 × 10^9^ L^−1^ and 0.2 × 10^9^ L^−1^, respectively) but recovered to normal levels at the 3‐month time‐point while the median absolute neutrophil counts did not recover to normal range until the 6‐month time‐point post‐alloHSCT (Figure 1d–f).

**Figure 2:**
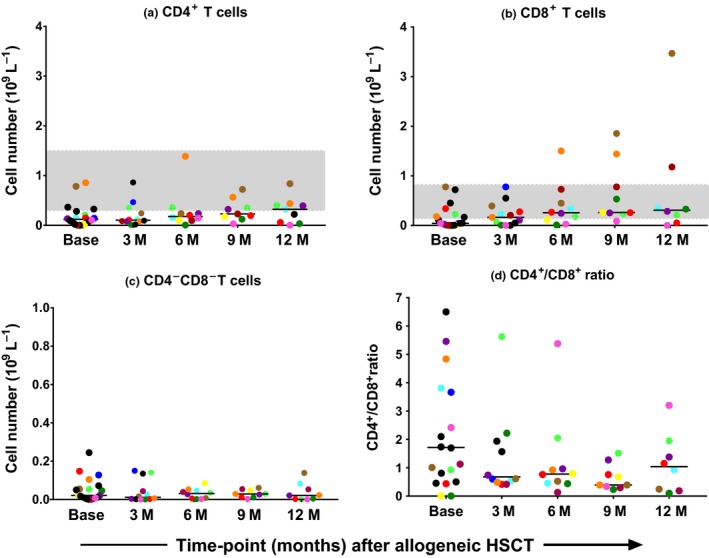
**(a–d)** Analysis of the T‐cell compartment for CD4^+^ and CD8^+^ absolute cell numbers and CD4^+^/CD8^+^ ratio. M, month; HSCT, haemopoietic stem cell transplant; *N* = 20 at time of conditioning chemotherapy (Base); *N* = 13 at 3 months (3 M); N = 11 at 6 months (6 M) and N = 9 at 9 months (9 M) and 12 months (12 M). The total WBC count was used to calculate absolute CD4^+^ and CD8^+^ T‐cells subsets based on flow cytometry immunophenotyping data (percent positive for each subset based on analysis of 100 000 acquired events). The grey bands depict the normal range for CD4^+^ and CD8^+^ T–cells. Black horizontal bars represent the median. Longitudinal samples from each patient are represented in individual colours. Data are representative of one experiment.

**Figure 3:**
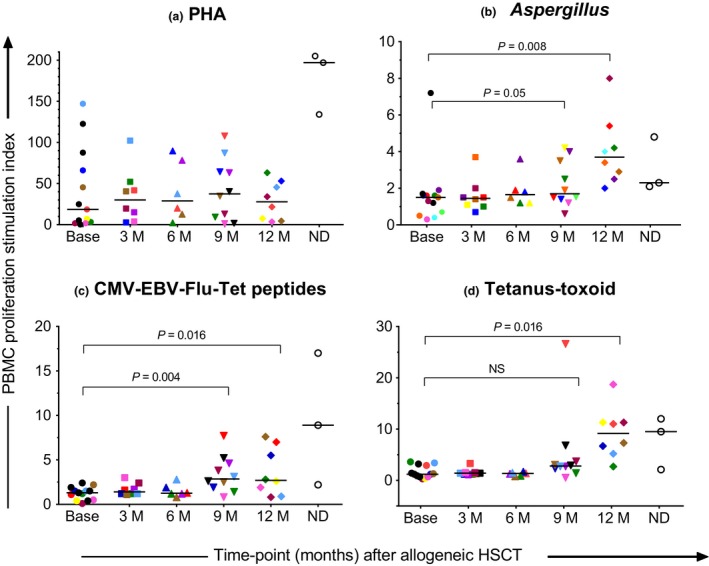
**(a–d)** Peripheral blood mononuclear cell responses to common antigens over 12 months of follow‐up. PHA, phytohaemagglutinin; M, month, ND, normal donor; CMV‐EBV‐Flu‐Tet peptide pool, cytomegalovirus, Epstein‐Barr virus, influenza, and tetanus peptides; HSCT, haemopoietic stem cell transplant; NS, not significant; *N* = 20 at time of conditioning chemotherapy (Base); *N* = 13 at 3 months (3 M); N = 11 at 6 months (6 M) and N = 9 at 9 months (9 M) and 12 months (12 M). Mean counts per minute with standard deviation (SD) were calculated for triplicate cultures (SD < 20% for all experiments). Black horizontal bars represent the median Stimulation Index (SI) for the patient group from one experiment for each patient sample. Longitudinal samples from each patient are represented by individual colours.

The median absolute count of CD4^+^ T cells was below lower limit of normal until the 12‐month time‐point (0.32 × 10^9^ L^−1^), while median absolute CD8^+^ T‐cell counts recovered to normal levels 3‐ to 6‐months post‐alloHSCT (Figures 2a and 3b). Patients 7, 11 and 17 who had high absolute CD8^+^ T‐cell counts after the 6‐month time‐point had multiple infections, and patient 17 also had extensive chronic GVHD (Figure 2b; Table 2). The median absolute CD4^+^ T‐cell counts in RIC‐alloHSCT recipients remained < 0.2 × 10^9^ L^−1^ (0.185 × 10^9^ L^−1^) at the 12‐month time‐point. Low numbers of CD4^‐^CD8^‐^ double negative T‐cells, not usually present in the periphery of healthy individuals, were seen in this cohort of alloHSCT recipients (Figure 2c). The CD4^+^:CD8^+^ T‐cell ratio was close to normal (1.7:1) at baseline but reversed during the 12 months of follow‐up (Figure 2d).

### Pathogen‐specific PBMC cell responses

T‐cell proliferative responses to *Aspergillus* lysate, tetanus‐toxoid and a peptide mix containing MHC Class II binding peptides from CMV, Epstein Bar virus (EBV), tetanus and Influenza (CMV‐EBV‐Flu‐Tet peptide pool) are shown in Figure 3a–d. The median SI for *Aspergillus*, tetanus‐toxoid and the CMV‐EBV‐Flu‐Tet peptide pool was < 2.5 (defined threshold) at baseline, indicating little to no capacity to proliferate. Positive proliferative responses were only observed 9‐ to 12‐months post‐alloHSCT for all antigens and peptides (Figure 3b–d). The median *Aspergillus* and CMV‐EBV‐Flu‐Tet peptide pool‐specific PBMC responses (Figure 3b, c) were statistically significantly higher at 9‐months (SI = 1.7 and 2.8, respectively) and 12‐months (SI = 3.7 and 2.7, respectively) post‐alloHSCT as compared with baseline (SI = 1.5 and 1.3, respectively) but tetanus‐toxoid‐specific proliferation (Figure 3d) was not statistically significantly higher until the 12‐month time‐point (SI = 1.2 vs. 9.2; *P *=* *0.016) post‐alloHSCT.

### Cytokine profile of pathogen‐specific T‐cells

**Figure 4:**
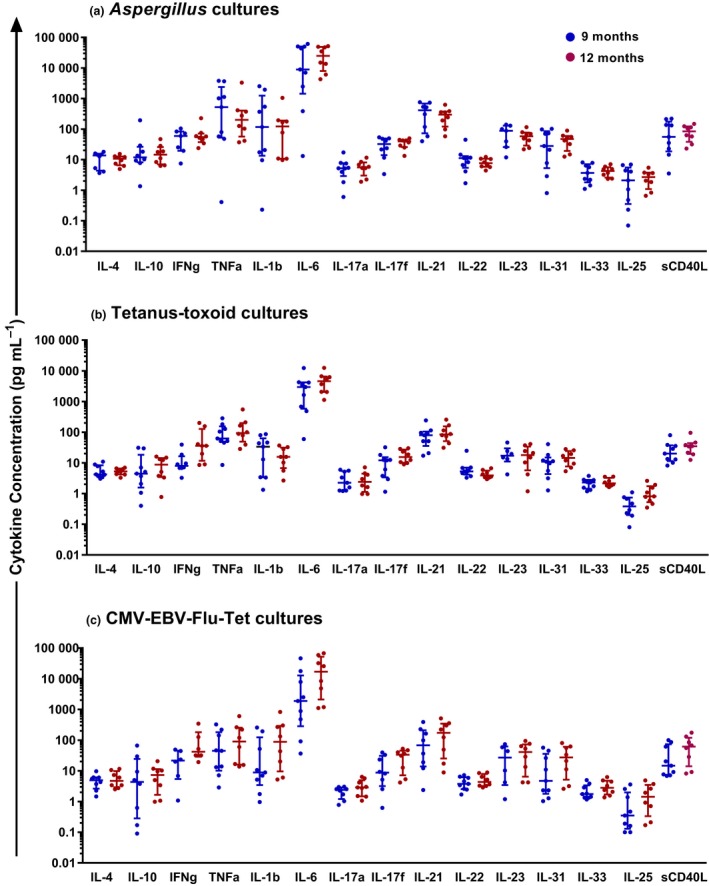
**(a–c)** Median antigen‐specific cytokine concentrations in culture supernatant of peripheral blood mononuclear cell cultures. CMV‐EBV‐Flu‐Tet peptide pool, cytomegalovirus, Epstein‐Barr virus, influenza, and tetanus peptides; HSCT, haemopoietic stem cell transplant; *N* = 9 at 9 months and 12 months. As significant proliferation was seen only at the 9‐ and 12‐month time‐points in *Aspergillus*, tetanus‐Toxoid and CMV‐EBV‐Flu‐Tet peptide pool cultures, we analysed cytokines only at these time‐points. The experiments were performed as singlicate assays and the antigen‐specific cytokine concentration shown is after background levels (cytokines secreted in the absence of antigens) have been subtracted for each patient sample. The blue and red horizontal bars represent the median, 25th and 75th percentiles of distribution. Data are representative of one experiment.

In the PBMC culture supernatants, cytokine levels were only analysed at the 9‐ and 12‐month time‐points, as significant proliferation was only detected at these time‐points. Median concentrations of cytokines secreted in response to *Aspergillus*, tetanus‐toxoid and the CMV‐EBV‐Flu‐Tet peptide pool are shown in Figure 4a–c. Cytokines representing Th‐1 (IFN‐γ), Th‐2 (IL‐4) and Th‐17 (IL‐17, IL‐21) subsets and others were detected indicating the presence of a broad T‐cell response which was not skewed towards a particular phenotype. Very high levels of IL‐6 (> 100 pg mL^−1^) were secreted in response to all antigens and peptides (Figure 4a–c). There was no significant difference between mean total cytokine levels between the 9‐ and 12‐month time‐points from *Aspergillus* (1943 vs. 1953 pg mL^−1^) and tetanus‐toxoid (253 vs. 358 pg mL^−1)^‐stimulated cultures (Figure 4a, b), but the mean total cytokine level in CMV‐EBV‐Flu‐Tet peptide‐stimulated cultures were significantly higher at 12 months as compared with 9 months (1742 vs. 655 pg mL^−1^; *P *=* *0.008; Figure 4c).

### Correlation between cell counts or cell percentages and pathogen‐specific cytokine secretion

Cytokine secretion following *Aspergillus* stimulation, showed that CD4^+^ T‐cell percent correlated with IL‐6 (*P *=* *0.043) secretion, while CD8^+^ T‐cell percent correlated with IL‐6 (*P *=* *0.03), IL‐17f (*P *=* *0.04), IL‐31 (*P *=* *0.037), IL‐33 (*P *=* *0.037) and IL‐25 (*P *=* *0.03) secretion.

The CD3^+^ T‐cell count showed significant correlation with the CMV‐EBV‐Flu‐Tet peptide pool‐specific secretion of IFN‐γ (*P *=* *0.016) and IL‐1β (*P *=* *0.013), as did CD4^+^ T‐cell counts with IL‐1β (*P *=* *0.045) secretion, while the CD8^+^ T‐cell count correlated with IFN‐γ (*P *=* *0.013) and IL‐1β (*P *=* *0.012) secretion. The NK‐cell count correlated with IL‐1β (*P *=* *0.02) and IL‐17a (*P *=* *0.03) secretion.

### Serum cytokines

**Figure 5:**
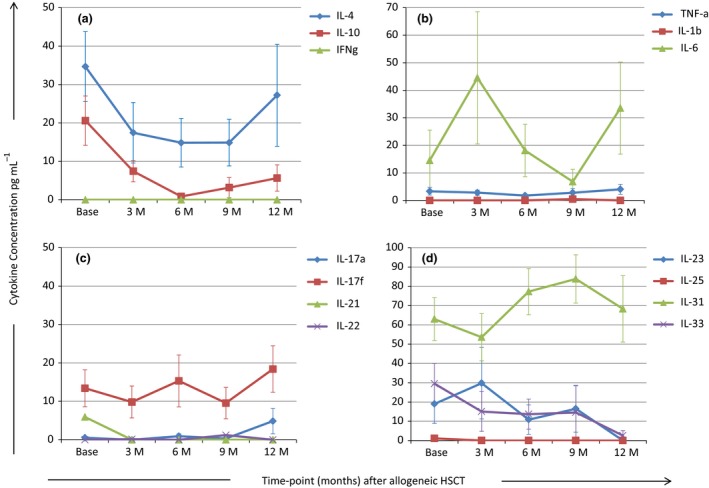
**(a–d)** Mean cytokine concentrations in serum over 12 Months of follow‐up after allogeneic haemopoietic stem cell transplant. M, month; IFN, interferon; TNF, tissue necrosis factor; HSCT, haemopoietic stem cell transplant; *N* = 20 at time of conditioning chemotherapy (Base); *N* = 13 at 3 months (3M); *N* = 11 at 6 months (6M) and *N* = 9 at 9 months (9M) and 12 months (12M). The experiments were performed as singlicate assays. Mean cytokine concentration is plotted for each time‐point and the vertical bars represent the standard error of the mean. Data are representative of one experiment.

The mean concentrations of serum cytokines (Figure 5a–d) were comparable with that reported for healthy donors in other studies.^19^, ^22^ IFN‐γ was not detected at any time‐point while the median levels of IL‐4 and IL‐10 decreased to reach their nadir at 6 to 9 and 6 months, respectively, and increased thereafter (Figure 5a). Two spikes of the pro‐inflammatory cytokine IL‐6 were detected at 3‐ and 12‐months post‐alloHSCT (Figure 5b). Median IL‐17a and Il‐17f levels were highest at 12 months (Figure 5c). IL‐31 reached the nadir at 3 months whereas IL‐23 and IL‐33 declined over the 12 months of follow‐up (Figure 5d).

### Antibody isotypes

**Figure 6:**
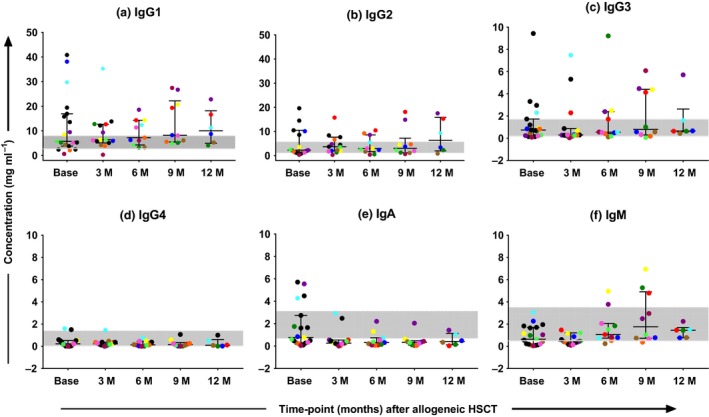
**(a–f)** Antibody isotypes in serum over 12 months of follow‐up after allogeneic haemopoietic stem cell transplant. Ig, immunoglobulin; M, month; HSCT, haemopoietic stem cell transplant; *N* = 20 at time of conditioning chemotherapy (Base); *N* = 13 at 3 months (3M); *N* = 11 at 6 months (6M) and *N* = 9 at 9 months (9M) and 12 months (12 M). Black horizontal bars represent the median, 25th and 75th percentiles of distribution. The grey band depicts the normal range.^32^, ^33^ Longitudinal samples from each patient are represented by individual colours. Data are representative of one experiment.

Median serum levels of IgG1, IgG2, IgG3, IgG4, IgA and IgM are shown in Figure 6a–f. The median concentrations of IgG1, IgG2 and IgG3 were above the lower limit of normal over the 12‐month follow‐up period (Figure 6a–c).^23^ Patients showing IgG1 and IgG3 levels above the normal range had no infections diagnosed at the corresponding time‐point. Median IgG1 (associated with Th‐1 responses) concentrations increased over time and were highest 12‐months post‐alloHSCT (Figure 6a). Median concentrations of IgG4 (associated with Th‐2 responses) remained at the lower limit of normal through the 12 months of follow‐up (Figure 6d).

The median concentrations of IgA were below the lower limit of normal throughout the follow‐up period (Figure 6e).^24^ Patients who had high IgA levels at baseline and at 3‐month post‐alloHSCT (patients 8, 10, 13 and 20) had mucosal infections diagnosed within 2 months of transplantation (Table 2). The median concentration of IgM was within the normal range throughout but highest at 9 months (Figure 6f).^24^

## Discussion

In the modern era of alloHSCT, this proof of concept study comprehensively measures the recovery of immune cell subsets, pathogen‐specific immune responses, cytokines and serum antibody isotypes. We report that recovery of functional CD4^+^ T‐cell pathogen responses occurs 9‐ to 12 month post‐alloHSCT and, based on antigen‐specific *in vitro* responses, identify a panel of cytokines (IFN‐γ, IL‐1β, IL‐4, IL‐6, IL‐17, IL‐21 and IL‐31), with potential for immune‐profiling and infection risk prediction.

As with other studies, we found that NK cell and monocyte recovery occurred before T‐cell and B‐cell recovery.^17^, ^25^ CD8^+^ T‐cells recovered earlier than CD4^+^ T‐cells, with the latter only reaching the reference range^21^ 12‐month post‐alloHSCT with reversal of the CD4^+^/CD8^+^ ratio (Figure 2a, b).^21^ This finding is likely due to viral reactivation and more efficient peripheral homeostatic expansion of CD8^+^ T cells.^11^ As CD4^+^ T cells play a central role in orchestrating all immune responses, CD4^+^ T‐cell counts and function are critical to infection risk post‐alloHSCT. Storek *et al*.^26^ demonstrated that long‐term infection morbidity is related to CD4^+^ T‐cell reconstitution post‐alloHSCT, and antigen‐specific responses were reduced if CD4^+^ T‐cell counts were low. Fukuda *et al*.^27^ demonstrated similar rates of invasive aspergillosis especially late, in RIC‐ as compared with myeloablative‐alloHSCT recipients. We have detected low CD4^+^ T‐cell counts at 12 months in our RIC‐alloHSCT recipients (median of 0.185 × 10^9^ L^−1^) and have demonstrated that recovery of CD4^+^ T‐cell count parallels *Aspergillus*‐specific T‐cell responses providing a potential explanation for the findings of Fukuda *et al*.^27^ In the HIV population, a CD4^+^ T‐cell count of < 0.2 × 10^9^ L^−1^ is used as a biomarker in guiding prophylaxis of the fungus *Pneumocystis jirovecii*.^28^ Regular measurement of CD4^+^ T‐cell counts post‐alloHSCT may have potential for guiding antimicrobial prophylaxis.

We found that median absolute neutrophil counts were below the reference range until 6‐months post‐alloHSCT. Rommeley *et al*.^29^ examined the recovery of neutrophil function in 30 alloHSCT recipients and demonstrated that 91.8% had recovered the capacity to phagocytose *Escherichia coli* but only 66.3% had recovered oxidative burst activity by Day 90 post‐alloHSCT. Further prospective evaluation of functional neutrophil recovery post‐alloHSCT is required.

As data on CD4^+^ T‐cell functional recovery post‐alloHSCT are limited, we assessed *in vitro* T‐cell proliferation using PBMC assays. While T‐cell subset percent fraction had returned to normal by 6 months, the recovery of absolute T‐cell numbers was delayed until 9‐months post‐alloHSCT. The detection of pathogen‐specific PBMC responses at 9‐months post‐alloHSCT (Figure 3) aligned closely with recovery of absolute T‐cell numbers (Figure 1). Lack of positive proliferative responses until 9‐months post‐alloHSCT is likely to be due to a combination of factors including low T‐cell numbers, ongoing immunosuppressive therapy or its residual effects and limited T‐cell repertoire diversity because of low thymic regeneration in adults post‐alloHSCT.^30^ Proliferative responses to phorbol‐12‐myristate‐13‐acetate‐Ionomycin were present at all time‐points (data not shown), indicating that signal transduction pathways were not affected by alloHSCT procedures. Current guidelines recommend that antifungal prophylaxis continues until Day 75 post‐alloHSCT (if no GVHD).^31^ However, our findings indicate that larger correlative studies should be performed to determine whether current guidelines should be revisited.

There are very limited data on *Aspergillus*‐specific T‐cell responses post‐alloHSCT. Perruccio *et al*.^32^ reported that *Aspergillus*‐specific immune recovery occurred in T‐cell depleted matched, T‐cell depleted haploidentical and T‐cell replete alloHSCT recipients at 7 to 9, 9 to 12 and 15 to 18 months, respectively. Beck *et al*.^33^ demonstrated that no significant *Aspergillus*‐specific T‐cell responses were detected in the first 4‐month post‐alloHSCT (*n* = 15), and in comparison with healthy controls (*n* = 23), *Aspergillus*‐specific T‐cell responses were significantly lower up to 1‐year post‐alloHSCT. Characterisation of *Aspergillus‐*specific T cells in humans has been challenging because of the low precursor frequency of fungal‐specific T cells seen in healthy individuals,^34^, ^35^ and the delayed recovery of *Aspergillus*‐specific responses we detected could be attributed to this. However, we found that tetanus‐toxoid and CMV‐EBV‐Flu‐Tet peptide pool responses recovered at similar time‐points post‐alloHSCT. Also, the timing of recovery of *Aspergillus‐*specific responses in our study was consistent with that of other studies.^32^, ^33^ Our findings indicate that recovery of *Aspergillus*‐specific T‐cell responses occur late post‐alloHSCT and may explain the previously reported late cases of invasive aspergillosis.^36^

The high tetanus‐toxoid‐specific proliferative responses seen at 12 months may be due to vaccination 4–6 months earlier (Supplementary table 1) or due to recovery of memory T‐cell responsiveness at this time‐point. Higher circulating CD4^+^ T‐cell counts have been reported to correlate with improved pneumococcal conjugate vaccine response.^14^ Comparatively, the proliferative response to CMV‐EBV‐Flu‐Tet peptide pool peptides is lower at 6 to 9 months which may be related to the dominance of CD8^+^ T cells seen in the CMV‐EBV‐Flu‐Tet peptide pool cultures which have lower proliferative capacity than CD4^+^ T cells. Although the major histocompatibility complex Class II binding CMV‐EBV‐Flu‐Tet peptide pool targets CD4^+^ T cells, it also contains cross‐reactive CD8^+^ T‐cell epitopes within its sequence. Avetisyan *et al*.^12^ reported that influenza T‐cell responses were broad with multiple epitopes and proteins recognised. Haining *et al*.^37^ showed, in vaccinated paediatric alloHSCT recipients, that CD4^+^ responses were stronger than CD8^+^ responses. Measurement of proliferative responses to both tetanus‐toxoid and CMV‐EBV‐Flu‐Tet peptide pool may have value from 6‐months post‐alloHSCT to better define the full spectrum of vaccine responsiveness and the optimal timing for re‐vaccination.

The cytokine secretion pattern of PBMC cultures demonstrated a broad range, including those representing Th‐1 (IFN‐γ), Th‐2 (IL‐4) and Th‐17 (IL‐17, IL‐21) subsets (Figure 4a–c). The cytokine profiles differed between *Aspergillus*, tetanus‐toxoid and CMV‐EBV‐Flu‐Tet peptide pool cultures. Further, the median cytokine concentrations were higher in *Aspergillus* and CMV‐EBV‐Flu‐Tet peptide pool cultures than in tetanus‐toxoid cultures (Figure 4). Nilsson *et al*.^38^ stimulated donor T cells with viral antigens and found that IL‐1β and IFN‐γ production was higher in grafts transplanted to recipients who had no post‐alloHSCT infectious complications compared to those who did. While further assessment is required, the data presented indicate that cytokine profiles may have potential in infection risk stratification to individualise antimicrobial prophylaxis post‐alloHSCT.

Even though median levels of proliferation was highest to tetanus‐toxoid (12 months), median cytokine secretion was lower compared to *Aspergillus* cultures, and no correlations were seen between T‐cell numbers and cytokine concentration in the tetanus‐toxoid cultures. The strong cytokine response generated by *Aspergillus* lysate is consistent with a polyclonal T‐cell response driven by a mix of proteins. Proliferation appears to be a better marker of tetanus‐specific immunity than cytokines reflecting the tetanus‐toxoid‐specific immune response which is predominantly antibody‐mediated and driven by Th‐2 rather than a cell‐mediated immune response.

The correlations observed between T‐cell and NK‐cell numbers and cytokine concentrations in the cultures suggest different cell sources for the various cytokines. In the *Aspergillus* cultures, IL‐6 levels correlated with NK‐, CD4^+^ T‐cell and CD8^+^ T‐cell numbers, IL‐25 with CD4^+^ and CD8^+^ T cells and IL‐17, IL‐31 and IL‐33 with CD8^+^ T cells. CD4^+^ T cells and NK cells are known to be important drivers of fungal‐specific immunity and Th‐17 cells play a central role in *Aspergillus* clearance.^39^, ^40^ Consistent with this, we detected a cytokine profile characterised by a Th‐17 driven *Aspergillus* response. IL‐6 is produced by a wide variety of cells including fibroblasts, keratinocytes, endothelial cells, monocytes, macrophages and T cells. As monocytes are possible contributors for IL‐6 detected in the PBMC cultures the correlations seen with T and NK cells could be indirect. The cytokine profile from the CMV‐EBV‐Flu‐Tet peptide pool stimulated cultures indicates IFN‐γ dominance from both CD8^+^ T cells and NK cells and CD8^+^ T cells are known to play a critical role in viral immunity post‐alloHSCT.^25^, ^41^ The CD8^+^ T‐cell contribution observed in *Aspergillus‐*stimulated cultures may reflect an adjuvant effect driving bystander CD8^+^ T‐cell activity in these CD4^+^ T‐cell deficient patients. Indeed, many of the patients in our cohort had viral coinfection (Table 2).

AlloHSCT is known to be associated with substantial changes in cytokine concentrations resulting from mucosal damage, consequent to the procedure. The proinflammatory cytokine IL‐6 was the most abundant cytokine in our PBMC cultures (Figure 4a–c). IL‐6 is known to play an important role in haematopoiesis and is required for initiation of Th‐17 responses.^42^ The cytokine profile is also impacted by the kinetics of cytokine secretion and as supernatant was taken at Day 4 from PBMC cultures, cytokines that are secreted earlier (IL‐6, TNF‐α, IL‐1β, IFN‐γ) are likely to be preferentially detected while those that peak after day 4 such as IL‐17 and IL‐22 may be under‐represented.^43^ Because the serum cytokine levels detected were low in our cohort (Figure 5) and are known to be highly variable even in healthy individuals,^19^, ^22^, ^44^ we would not advocate their measurement in clinical practice.

Serum antibody isotyping in our patient cohort (Figure 6a–f) showed that the two most abundant subclasses, IgG1 and IgG2, were within the normal range even before B‐cell numbers recovered, suggesting functional long‐lived plasma cells. Patients who had high levels of IgG3 which is most efficient at complement activation and opsonisation did not have active infections at the time, implying a protective function. In contrast to the other subclasses, IgG4 which is noncomplement activating and known to be produced in response to allergens was low throughout the 12‐month period. The stronger IgG1 and low IgG4 responses in this cohort maybe suggestive of a Th‐1 bias post‐HSCT.^45^ Although high IgA levels were detected in patients with mucosal infections in the early time‐points, median levels were below normal up to 12‐months post‐alloHSCT. The low levels of some antibody isotypes at the 9‐ to 12‐month time‐point are suggestive of defective Ig‐class switching in this cohort. The spike in IgM concentration at 9‐months post‐alloHSCT may reflect vaccinations introduced around 6 to 8 months.

Our finding of high concentrations of IL‐31 in PBMC cultures and serum is novel in the alloHSCT setting. IL‐31 is produced by activated Th‐2 type T cells, induces production of IL‐6,^41^ and has been shown to be associated with inflammatory disorders. IL‐33, which is produced by necrotic and epithelial cells and has increased function under cellular stress, was seen predominantly in our *Aspergillus* antigen re‐stimulation cultures. IL‐33 is known to drive Th‐2 responses and play a crucial role in allergy and lung‐related inflammatory conditions.^44^ Our novel findings in relation to IL‐31 and IL‐33 warrant further investigation in a larger cohort of alloHSCT recipients as these may serve as biomarkers that can be used for risk prediction of *Aspergillus* infections post‐alloHSCT.

Limitations in the present study include the small sample size restricted 15‐plex cytokine analysis and the narrow antigen range used to assess immune responses. The small sample size prohibited us from effectively determining cause‐effect associations, and the correlations observed need to be interpreted with caution. However, this was a pilot study designed to assess feasibility of detecting potential immune biomarkers for improved infection risk prediction post‐alloHSCT. The findings provide a framework for larger cohort studies where immune responses to a broader range of vaccine antigens and opportunistic pathogens can systematically be measured along with extensive analysis of cytokine profiles. In addition, PBMC assays have many limitations including lengthy purification process and assay time, and cell loss from cryopreservation. The recently developed flow‐based assay using whole blood has the advantage of combining immunophenotyping with detection of antigen‐specific proliferation and cytokine secretion (FASCIA). This rapid and practical assay has demonstrated capacity to detect lymphocyte reactivity in primary immunodeficiency and HIV settings.^19^, ^46^ Of note, Nilsson *et al*.^38^ used FASCIA to determine immune function in donor grafts to predict infection risk post‐alloHSCT.

In conclusion, this proof of concept study indicates that functional immune reconstitution post‐alloHSCT can be measured. We have identified CD4^+^ T‐cell counts, antigen‐specific T‐cell proliferation and cytokine production (IFN‐γ, IL‐1β, IL‐4, IL‐6, IL‐17, IL‐21 and IL‐31), in response to a variety of opportunistic and vaccine‐preventable pathogens, as having potential to be utilised as biomarkers for infection risk prediction. Larger multicentre studies are required for validation and to fully elucidate the clinical utility of these immune assays.

## Methods

### Patients

Twenty patients aged 18 years or older who underwent alloHSCT at Alfred Health, Melbourne, Australia, between February 2015 and January 2016 were enrolled and followed for 12 months or until death, if earlier. Written informed consent was obtained from all participants within 96 h of commencing conditioning chemotherapy. The study was approved by Alfred Health Human Research Ethics Committee (344.14). All procedures of alloHSCT including immunosuppression regimens, blood product support, and the investigation and treatment of transplant complications were as per standard Alfred Health protocols. All suspected infections were actively investigated using cultures (e.g. blood, urine), radiology (e.g. computed tomography scan), scopes (e.g. bronchoscopy, colonoscopy), tissue biopsy (e.g. open lung biopsy) and blood for molecular and serological testing (e.g. CMV, respiratory viruses, *Legionella*), as previously described.^47^

### Study definitions

Cytomegalovirus infection and disease were defined according to published criteria.^48^ The European Organization for the Research and Treatment of Cancer/Mycoses Study Group criteria were used to classify invasive fungal disease invasive fungal disease.^49^ Published definitions were used to classify bacterial infections and infection‐related mortality.^50^ Respiratory viral infections were defined as positive PCR results from respiratory specimens together with characteristic respiratory symptoms.^51^ Polyomavirus and norovirus infections were defined as positive PCR from blood and disease as detection of virus by PCR or immunohistochemistry in bodily fluids or organ tissue together with symptoms and/or signs in the affected organ.^52^, ^53^ The guidelines of Sarmati *et al*.^54^ were used to identify cases of Hepatitis B infection. Acute and chronic GVHD were classified according to the Glucksberg and the National Institute of Health criteria, respectively.^55^, ^56^

### Sample preparation

Whole blood and serum were collected from 20 patients at baseline (within 96 h of commencement of conditioning). Follow‐up samples were collected from 13, 11, 9 and 9 patients at 3‐, 6‐, 9‐ and 12‐months post‐alloHSCT, respectively. Peripheral blood mononuclear cells (PBMC) were purified by density gradient centrifugation with Ficoll‐Paque (GE Healthcare, USA) according to standard procedures. Briefly, whole blood was diluted 1:1 in sterile PBS and 30 mL layered on to a 15 mL gradient of Ficoll‐Paque and centrifuged for 20 min at 400* g*. The cell interface layer was carefully harvested and washed twice in PBS (10 min at 500 *g*) and the pellet resuspended in RPMI 1640 with Glutamax (Invitrogen, USA) and supplemented with 10% (v/v) Human AB serum (Sigma‐Aldrich, USA) and 100 U mL^−1^ Penicillin/Streptomycin solution (Invitrogen).

### Flow cytometric analysis of cell subsets

The 8‐colour antibody panel, CD3‐FITC, CD19‐APC, CD14‐APC‐H7, CD45‐PerCP‐Cy5.5, CD8‐BV421, CD4‐PE‐Cy7, CD15‐BV650 and CD16^+^CD56‐PE (BD Biosciences, USA), was used for immunophenotyping. Briefly, 100 μL of whole blood was stained with the antibody‐conjugate panel, red cells lysed and washed according to manufacturer's instructions and analysed on a BD LSR II flow cytometer (BD Biosciences). Cells were gated using forward and side scatter, doublets excluded, CD45^+^ gate applied for leucocytes and > 100 000 events collected. Rainbow Calibration Particles with six peaks (BD Biosciences) were used for quality control. The absolute cell number for each subset was calculated by multiplying total leucocyte count for each patient by percent fraction of each cell subset obtained immunophenotyping.

### PBMC assays for pathogen‐specific proliferative responses

To detect T‐cell responses, PBMC (1 × 10^5^) were cultured with *Aspergillus fumigatus* extract (Allergon, Angelholm, Sweden), tetanus‐toxoid (Statens Serum Institute, Denmark) and the MHC Class II peptide mix incorporating peptides from CMV, EBV, influenza and tetanus (CMV‐EBV‐Flu‐Tet peptide pool; JPT Peptide Technologies, Berlin, Germany). Phytohaemagglutinin (PHA; Sigma, MO, USA) was included as the positive control and PBMC alone in the absence of antigen included to assess background levels of proliferation. After 4 days 30 μL of supernatant was removed from each culture and stored at −80°C for cytokine analysis. The PBMC cultures were then supplemented with 30 μL fresh media containing 1 μCi of methyl‐^3^H Thymidine (^3^HTdR; MP Biomedicals, Australia) and harvested 16–20 h later. Incorporation of ^3^HTdR was measured by liquid scintillation spectroscopy as counts per minute (cpm), and mean counts per minute with standard deviation (SD) was calculated for triplicate cultures (SD < 20% for all experiments). Results are expressed as Stimulation Index (SI) which is the ratio of cpm for proliferation in presence of antigen/proliferation in PBMC alone culture and an SI > 2.5 was defined as a positive antigen‐specific response.

### Multiplex enzyme‐linked immunosorbent assay (ELISA) for cytokines in culture supernatant and serum

Cytokines, interleukin‐1β (IL‐1β), interferon‐gamma (IFN‐γ), tissue necrosis factor‐alpha (TNF‐α), IL‐4, IL‐6, IL‐10, IL‐17a, IL‐17f, IL‐21, IL‐22, IL‐23, IL‐25, IL‐31, IL‐33 and sCD40L was assessed in singlicate assays in serum and culture supernatants from antigen‐stimulated cultures, using the Bio‐Plex Pro^™^ Human Th‐17 15‐Plex assay (Bio‐Rad Corporation, CA, USA), according to manufacturer's instructions with cytokine concentrations automatically generated by Bio‐Plex 200 system software. The reference range for healthy adult serum in this assay reported by the manufacturer is IL‐4: 0.06‐3.0; IL‐10: 0.4‐2.0; IFN‐γ: 7.0‐124.0; TNF‐α: 6.0‐98.0; IL‐1β: < 0.7; IL‐6: 0.5‐9.0 and IL‐17: 2.0‐31.0 pg mL^−1^. No data are available for the normal range in serum for IL‐21, IL‐22, IL‐23, IL‐25, IL‐31 and IL‐33 by this assay. For culture supernatants, we included supernatants from PHA stimulated PBMC cultures from each patient as the positive control. We also tested cytokine secretion from PBMC in the absence of any antigen (background control). The values shown are antigen‐specific cytokine secretion after background levels were subtracted

### Multiplex ELISA for antibody (immunoglobulin; Ig) isotyping

Antibody isotypes IgG_1_, IgG_2_, IgG_3_, IgG_4_, IgA and IgM in serum were assessed in singlicate using the Bio‐Plex Pro^™^ Human Isotyping Assay (Bio‐Rad Corporation) according to manufacturer's instructions and analysed on the Bio‐Plex 200 system.

### Statistical analysis

Descriptive statistics including proportion, mean ± standard deviation (SD), mean ± standard error of the mean (SEM), median, range and interquartile range (IQR) were calculated. The Mann–Whitney *U*‐test was used for comparison of continuous variables. Correlations were performed using Pearson's correlation coefficient. A *P*‐value of < 0.05 was considered statistically significant. Statistical analysis was performed using GraphPad Prism software (San Diego, CA, USA).

## Conflict of interest

The authors declare no conflict of interest.
