# Supplementary figures and images for: Contemporary analysis of functional immune recovery to opportunistic and vaccine‐preventable infections after allogeneic haemopoietic stem cell transplantation
Source: Clin Transl Immunology. 2018 Oct 5;7(10):e1040. doi: 10.1002/cti2.1040 (PMC6173278; doi:10.1002/cti2.1040)

(a) T cells

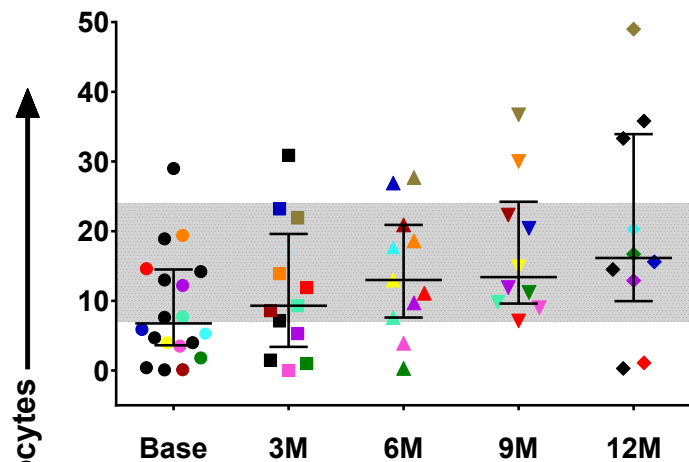

(b) B cells

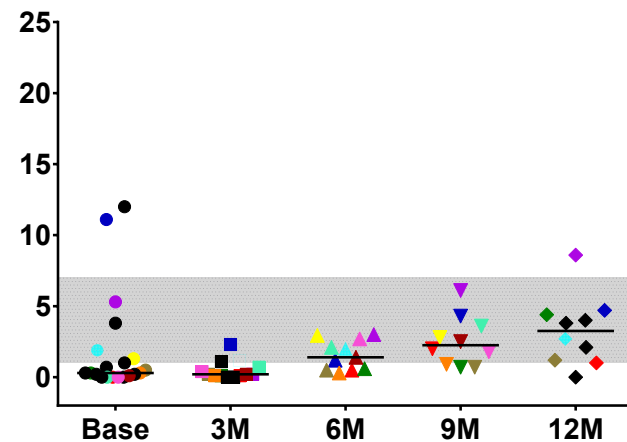

(c) NK cells

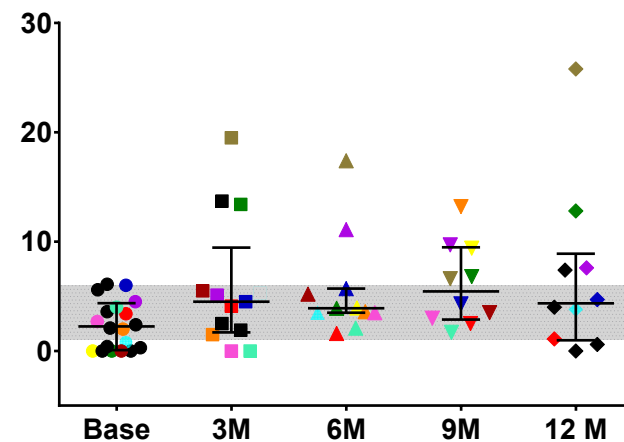

(d) Monocytes

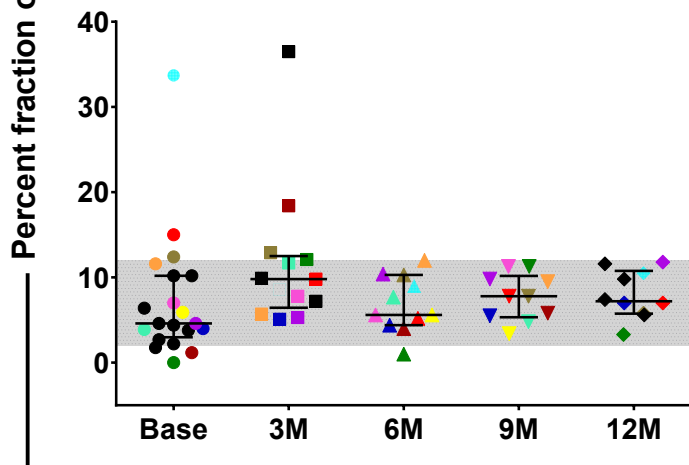

(e) Neutrophils

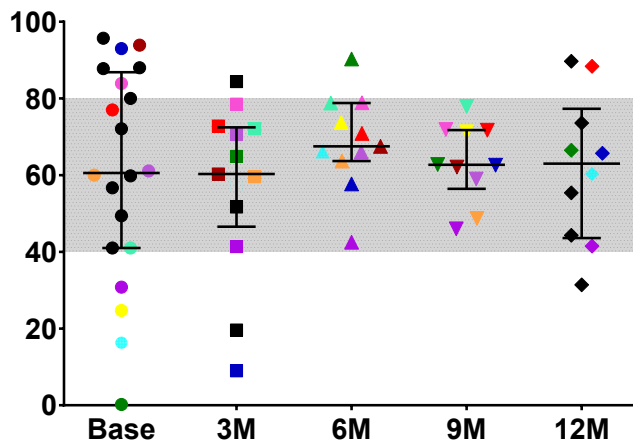

Time-point (months) after allogeneic HSCT

Supplement: Supplementary file 1 [file CTI2-7-e1040-s001.pdf]
