# Supplementary material for: Contemporary analysis of functional immune recovery to opportunistic and vaccine‐preventable infections after allogeneic haemopoietic stem cell transplantation
Source: Clin Transl Immunology. 2018 Oct 5;7(10):e1040. doi: 10.1002/cti2.1040 (PMC6173278; doi:10.1002/cti2.1040)
Supplement: Supplementary file 2 [file CTI2-7-e1040-s002.docx]

**Supplementary Table 1. Immune status and re-vaccination schedule through 1-year of follow-up**

| Patient | Revaccination Status | CD4+ T-cell Counts at 9-months post-alloHSCT  (x 10^9^ cells L^-1^)* | T-cell responses  9 months post-alloHSCT (SI)  Tet-Tox CEFT | | **Time of Revaccination (Month Post-HSCT)** | | | | | |
| --- | --- | --- | --- | --- | --- | --- | --- | --- | --- | --- |
|  |  |  |  |  | Diphtheria, Tetanus, Pertussis, Poliomyelitis | *Streptococcus pneumoniae* | *Haemophilus influenzae* type B | *Meningococcus* (ACWY strains) | Hepatitis B  (Detected Antibody response) | Influenza |
| 1 | Yes | 0.195 | 26.6 | 7.7 | 8 | 8 | 8 | 8 | 8 (26 mIU/ml |  |
| 2 | Yes | NA | 2.3 | 1.9 | 12 | 12 | 12 | 8 | 12 (ND) |  |
| 3 | No (unwell) | 0.122 | 1.4 | 1.4 |  |  |  |  |  |  |
| 4 | No (died) |  |  |  |  |  |  |  |  |  |
| 5 | Yes | 0.176 | 2.7 | 4.6 | 12 | 12 | 12 |  | 12 (37 mIU/ml) | 11 |
| 6 | No (died) |  |  |  |  |  |  |  |  |  |
| 7 | Yes | 0.722 | 3 | 2.5 | 6 | 6 | 6 | 6 | 6 (prior infection) |  |
| 8 | Yes | 0.322 | 2.7 | 3.1 | 8 | 8 | 8 | 8 | 8 (ND) |  |
| 9 | No (died) |  |  |  |  |  |  |  |  |  |
| 10 | No (LFTU) |  |  |  |  |  |  |  |  |  |
| 11 | Yes | 0.564 | 3.7 | 3.8 | 8 | 8 | 8 | 8 | 8 (ND) |  |
| 12 | No (died) |  |  |  |  |  |  |  |  |  |
| 13 | Yes | NA | NA | NA | 8 |  | 8 | 8 | 8 (22 mIU/ml) | 6 |
| 14 | No (died) |  |  |  |  |  |  |  |  |  |
| 15 | No (died) |  |  |  |  |  |  |  |  |  |
| 16 | No (died) |  |  |  |  |  |  |  |  |  |
| 17 | Yes | 0.228 | 0.5 | 0.8 | 12 |  | 12 | 8 | 12 (0 mIU/ml) |  |
| 18 | Yes | 0.350 | 2.9 | 2.6 | 8 | 8 | 8 | 8 | 8 (16 mIU/ml) | 6 |
| 19 | No (unknown) | 0.029 | 6.8 | 5.2 |  |  |  |  |  |  |
| 20 | No (died) |  |  |  |  |  |  |  |  |  |

Allo, allogeneic; HSCT, hematopoietic stem cell transplant; SI, stimulation index; Tet-Tox, Tetanus-toxoid ; CEFT, Cytomegalovirus, Epstein-Barr Virus, Influenza Virus and Tetanus peptide mix; NA, not available; ND, not determined; LFTU, lost to follow up (last visit – 3 months)

*normal range for CD4 + T cell count is 0.3 – 1.5 x 10^9^ cells L^-1^
